# Dimensionality, psychometric properties, and population-based norms of the Turkish version of the Chalder Fatigue Scale among adults

**DOI:** 10.1186/s12955-022-02074-x

**Published:** 2022-12-07

**Authors:** Rıdvan M. Adın, Ali Naim Ceren, Yeliz Salcı, Ayla Fil Balkan, Kadriye Armutlu, Çiğdem Ayhan Kuru

**Affiliations:** Faculty of Physical Therapy and Rehabilitation, Hacettepe University, Ankara, Turkey

**Keywords:** Fatigue, Public health, Quality of life, Reliability, Validity, Translation

## Abstract

**Background:**

Fatigue is emerging as a major public health problem that is highly associated with poor health-related quality of life and disability. Among adults, fatigue has become increasingly common because of workload or lifestyle changes. This study aimed to cross-culturally adapt the Chalder Fatigue Scale (CFS) into Turkish, to investigate its psychometric properties, and to establish normative data in healthy adults by age and gender.

**Methods:**

The validity of the CFS was tested with a total sample of 476 healthy adults aged 20–40 years (264 males and 212 females) and test–retest/measurement error analyses were performed with 161 participants (94 males and 67 females). The test–retest reliability was examined using the intraclass correlation coefficient (ICC), and internal consistency was determined using Cronbach's α-coefficient. Predictive validity was assessed using the Receiver Operating Characteristic to validate the cut-off value of the CFS for non-fatigued and fatigued participants. Factor analyses and hypothesis testing were conducted to assess construct validity. Hypothesis testing examined convergent and known-group validity by testing 14 predefined hypotheses.

**Results:**

The mean (SD) and median (25–75%) CFS scores were 10.7 (4.9) and 11 (7–14) for the total sample (n = 476). The cut-off point for CFS was set at ≥ 12 with a sensitivity of 65.8% and a specificity of 85.9%. The CFS provided evidence of excellent fit of the two-factor structure (CFI = 0.963, RMSEA = 0.06, SRMR = 0.02). There was evidence of strong internal consistency demonstrated by Cronbach's α = 0.863 and good test–retest reliability by ICC = 0.76. Thirteen out of 14 hypotheses (92.9%) were confirmed and the scale showed low to moderate correlation with other measurement instruments (r = 0.31–0.51).

**Conclusions:**

The CFS has been shown to be a reliable and valid instrument that can be used in various populations for the assessment of fatigue.

**Level of evidence:**

Level II.

## Background

Fatigue is a global phenomenon that negatively affects the biological, psychological, and cognitive processes of individuals. This distressing feeling is one of the most commonly reported symptoms to healthcare professionals [1, 2]. In several areas, fatigue remains an important issue that affects individuals’ health-related quality of life, employee health, safety, and overall work productivity by 54% [3, 4]. Recently, it has become a major concern for the physical and mental health of individuals working under challenging workload conditions during the COVID-19 pandemic [2, 5–7]. The prevalence of fatigue among patients who recovered from COVID-19 ranges from 52 to 70% [8, 9]. The assessment of fatigue is therefore becoming increasingly important in both clinical and healthy populations.

Several self-reported fatigue scales are used to evaluate the severity and characteristics of fatigue, and they have some advantages and disadvantages when compared with each other [10]. The Chalder Fatigue Scale (CFS) is an easy-to-understand, brief, and useful scale for individuals. The CFS was developed to measure the severity of perceived fatigue, which consists of two dimensions, including physical and mental fatigue [11, 12]. The measurement properties of the CFS have been studied in the general population and various disease groups but not in healthy adults [11–18].

Physical and mental fatigue affects adversely all segments of the population. In young adults, fatigue increases significantly due to various factors such as lifestyle, occupational overload, or socio-relational difficulties [19–22]. Population-based norms are needed to determine the prevalence of comorbidity in such a population. However there are few studies addressing this disturbing feeling in healthy young adults [19, 20, 23], and no study has established normative CFS scores in adults by sex or age. The first aim of this study was to cross-culturally adapt the CFS into Turkish and examine its psychometric properties, including criterion validity, construct validity, internal consistency, test–retest reliability, and measurement error. Second, we aimed to establish normative data in healthy adults by age and gender.

## Methods

### Study design

This study was designed as a methodological study conducted between May 2019 and October 2022. The study was approved by the Non-interventional Clinical Research Ethics Committee of Hacettepe University (May 14, 2019, GO 19/512). The study was conducted in two phases. In the first phase, the cross-cultural adaptation of the CFS was performed by following the guidelines provided by Beaton et al. [24]. In the second phase, the psychometric properties of the CFS -Turkish were evaluated in healthy adults according to the COSMIN criteria (COnsensus-based Standards for the selection of health Measurement INstruments) [25].

### Phase 1: The translation process

**Fig. 1:**
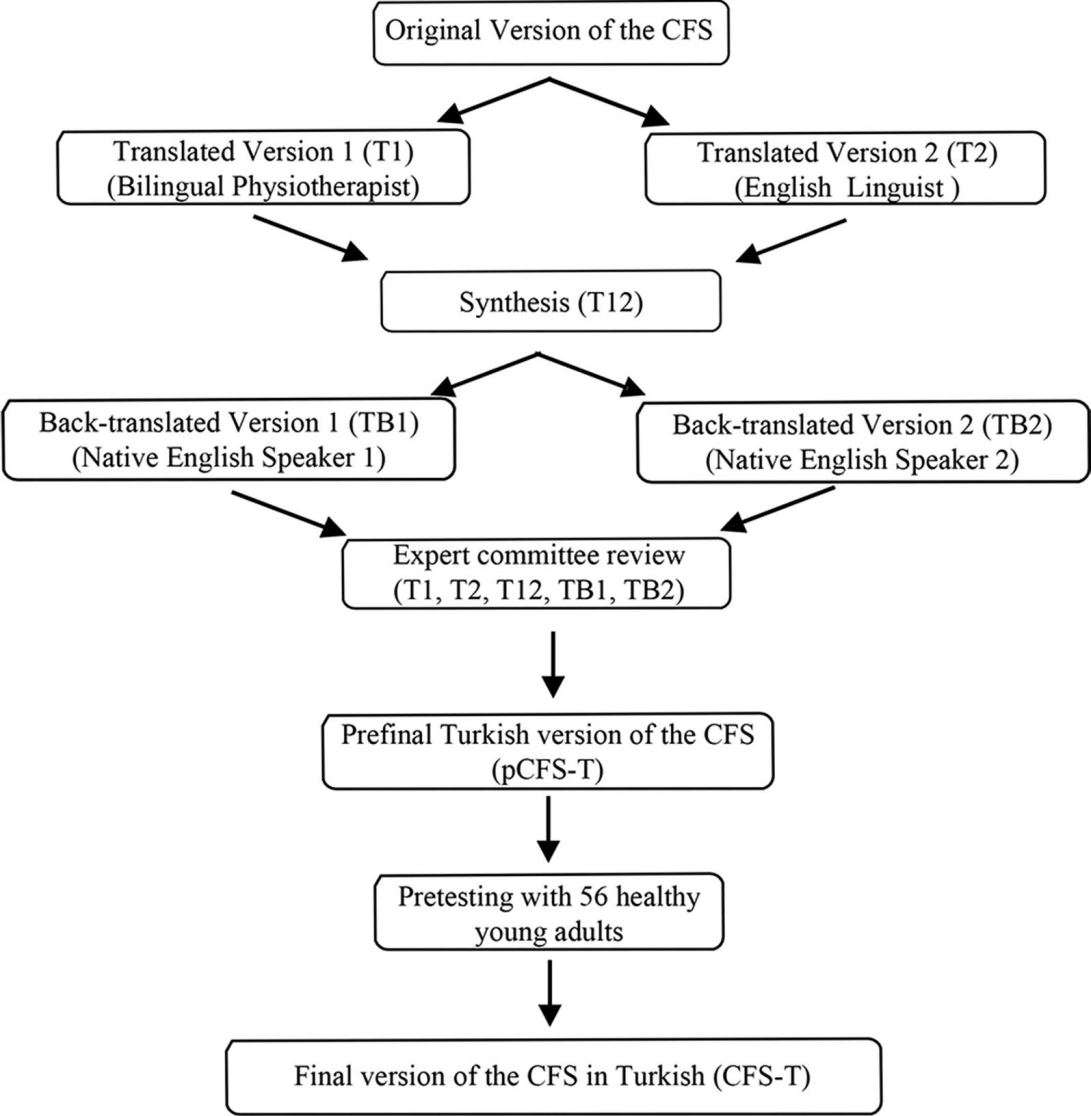
Translation process of the Turkish version of the Chalder Fatigue Scale

The translation of the CFS into Turkish was completed in six steps (Fig. 1) [24].

*Step 1-Translation*: Two bilingual Turkish translators were involved in the first step: a physiotherapist who is familiar with the instrument and an English linguist who has no medical background and is not familiar with the instrument. The translation of the CFS from English to Turkish was performed independently (T1 and T2).

*Step 2-Synthesis*: The two translated versions were compared and synthesized into one translated version (T12).

*Step 3-Back-translation*: Two native English speakers, fluent in Turkish and unfamiliar with the original instrument, performed the back translation of the T12 from Turkish into English and developed two new versions of the scale (TB1 and TB2).

*Step 4-Expert committee review:* An expert committee of researchers and translators compared the original CFS with the five translated versions (T1, T2, T12, TB1, TB2) to determine if all instruments were similar. After an agreement, the committee developed the prefinal Turkish version of the CFS (pCFS-T).

*Step 5-Pretesting:* The pCFS-T was tested on 56 volunteer healthy young adults (not included in the study sample). The participants rated the comprehensibility of items based on a three-point Likert scale (clearly/partially/not understandable). This strategy aimed to establish whether the pCFS-T was intelligible for this population. After the pretesting, it was determined that all participants rated all items in the scale as ‘clearly understandable’, which indicated that the pCFS-T was appropriate for this population.

*Step 6- Final Version:* All reports and forms were confirmed by the original developer. Consequently, the pCFS -T was introduced in its final version (CFS-T).

### Phase 2: Evaluation of the psychometric properties

#### Sample and data collection

Healthy young adults aged 20–40 years, who could read and speak Turkish, were included in the study. Exclusion criteria included acute/chronic illness, a surgical procedure in the past 6 months, use of prescribed or over-the-counter medications or supplements, < 17 kg/m^2^ body mass index > 30 kg/m^2^, depression with a score of ≥ 21 on the Beck Depression Scale (BDS), and pain with a score of > 0 on the pain subscale of the Nottingham Health Profile (NHP). Female participants were also excluded if they suffered from premenstrual syndrome or had been pregnant within the past year.

The sample size for reliability and validity analysis was determined in accordance with the literature [26–28]. A sample size of at least 200 individuals is required for validation studies and a sample size of at least 50 individuals per group is required for known group validity studies [26, 27]. For the analysis of internal consistency and test–retest reliability, a sample size of at least 100 and 30 individuals, respectively, is recommended [27, 28].

*Test Group:* We used snowball sampling, starting with 20 individuals (primary seeds) from all regions of Turkey. A research file (measurement instruments with written instructions and an introductory letter) was distributed in envelopes in person or by post to the authors' acquaintances who agreed to participate, and they were asked to invite others they knew who met the inclusion criteria and could participate in the study. All participants were informed about the study in advance and provided written informed consent. In all cases, participants received written instructions and an introductory letter explaining the scope of the study ("the study in which you will participate aims to assess the CFS in our culture"). The envelopes of the research files were returned in person or by post.

**Fig. 2:**
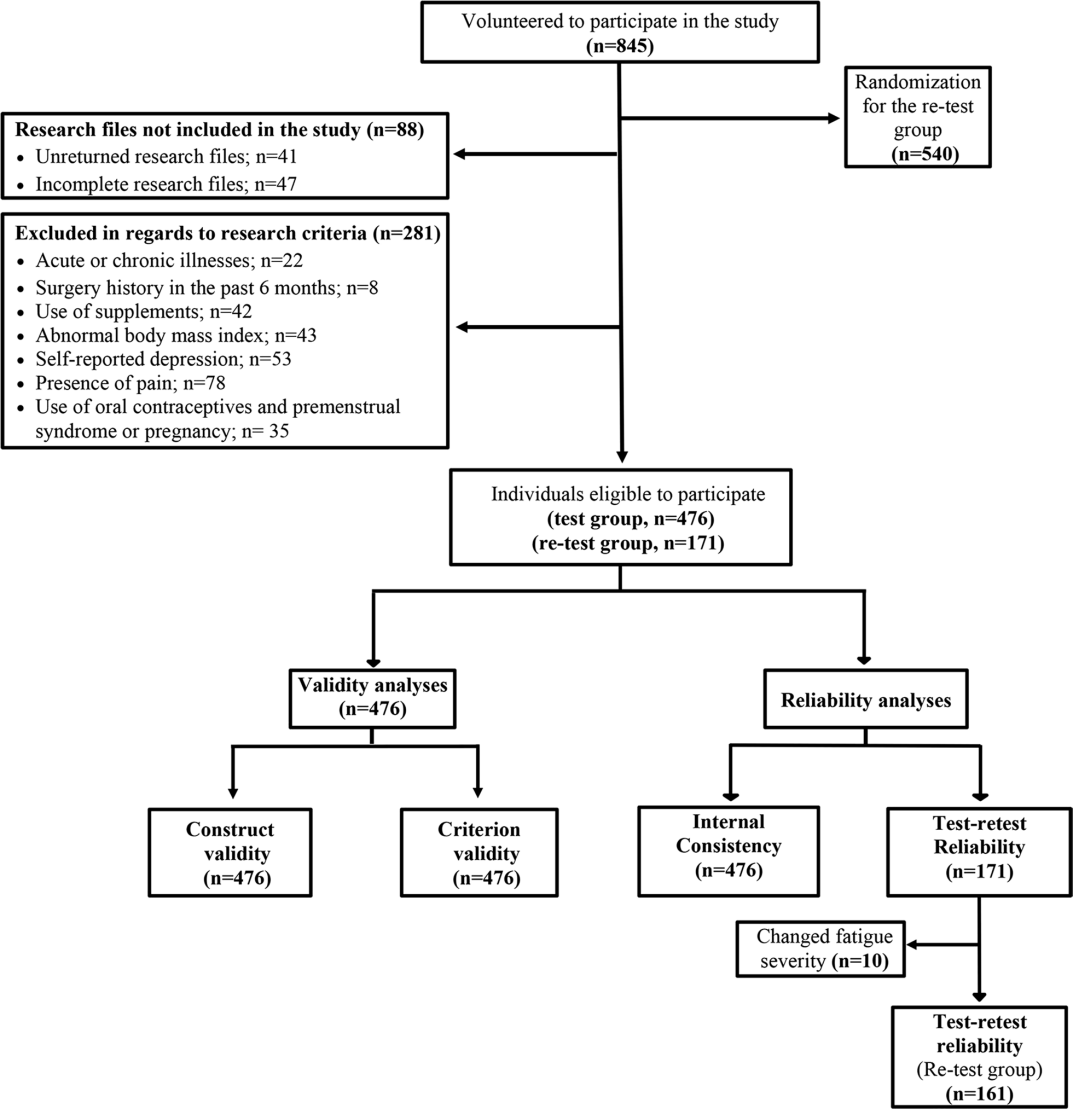
Flow diagram of the study

Special care was taken to ensure that the number of participants was representative of the entire age range of young adults and that the distribution of both genders was representative. Eight hundred and seventy participants were invited and 845 volunteers participated in the study. Of the 845 volunteers, 41 did not return their research file (real response rate: 801/870, 92.1%) and 47 did not complete measurement instruments properly (see missing item analysis). Based on the exclusion criteria, 281 individuals were excluded from the study. A total of 476 individuals participated in the initial assessment (Fig. 2).

*Re-test Group:* To investigate test–retest reliability, 540 of 845 volunteers were randomized using the Statistical Package for Social Sciences (SPSS) version 26.0 (SPSS Inc., Chicago, Illinois, USA) before sample selection. This resulted in 161 individuals being included in the reassessment survey based on the research criteria. The re-test group completed the CFS within 3–7 days. To minimize diurnal variation in fatigue levels, the reassessment was administered at the same time of day as the initial assessment [29].

The present study reached a sufficient sample size with 476 participants for the validity and internal consistency analyses and 161 participants for the test–retest and measurement error analysis. The flowchart of the study is shown in Fig. 2.

*Missing item analysis:* The quality of the data collected was checked for each outcome measure. Participants who did not answer a particular outcome measure or more than 10% of the total variables (110 items for validity, 11 items for internal consistency, 22 items for reliability analyses) were excluded (n = 47) from the study (Fig. 2). Missing values that were present in the research data were analyzed and imputed using SPSS as medians of the corresponding items. Incidental missing values were 2.8% (1466 of 52360 (110 × 476) items), 2.2% (115 of 5236 (11 × 476) items), and 2.9% (102 of 3542 (22 × 161) items) for the validity, internal consistency, and test–retest/measurement error analysis, respectively. Due to the small percentage of the missing data (< 3%), the imputation of the missing items did not significantly affect the results of the study [30].

### Measurements

All participants completed the CFS, Checklist Individual Strength (CIS), Visual Analogue Scale (VAS), NHP, BDS, and Pittsburg Sleep Quality Index (PSQI) at the initial assessment [31–35]. Participants were asked to complete sociodemographic profile information, including age, gender, weight, height, marital status, type of employment, working hours per week, education level, and usage of prescribed or over-the-counter medications/nutritional supplements.

*Chalder Fatigue Scale:* The 14-item scale (CFS-14) was first developed in 1993 to assess perceived fatigue [11]. In 2010, a revised version (CFS-11) of the original scale was published with three items removed [12]. The final version of the CFS with 11 items consists of 2 subscales: physical fatigue (CFS-PF) and mental fatigue (CFS-MF), and it can be rated on 2 different methods (bi-modal scoring {0–1} and 4-point Likert scoring {0–3}) [12]. The score of the CFS-PF ranges from 0–7, 0–21; the CFS-MF ranges from 0–4, 0–12, and the CFS-total ranges from 0–11, 0–33 points on the bi-modal and Likert scoring systems, respectively. Lower scores indicate a low level of fatigue [12]. Individuals with a total score of ≥ 4 are identified to be severely fatigued in bi-modal scoring and this scoring system is used in epidemiological studies [11, 15]. The CFS is available in 7 languages: Portuguese [36], Chinese [13], Korean [14], Norwegian [15], Japanese [16], Dutch [37], and Polish [38]. Its validity and reliability have been demonstrated in chronic fatigue syndrome [12, 39], multiple sclerosis [17], hemodialysis [40], primary care patients [36], pregnant women [38], and the general population [12–15]. The CFS has been found to be reliable (Cronbach's α for the subscales = 0.72–0.87 and for the CFS = 0.73–0.89) and to be valid in several studies [12, 13, 36, 37].

*Checklist Individual Strength:* The CIS consists of a total of 20 items and four subscales: subjective perception of fatigue (CIS-FS) (8 items), concentration (CIS-C) (5 items), motivation (4 items), and physical activity (3 items). The total score ranges from 20 to 140 and high scores indicate severe fatigue, low motivation, low concentration, and low physical activity [31]. The CIS is found to be a valid and reliable scale for assessing fatigue in healthy adults [10, 41]. The Turkish version of the scale was showed to have validity and good reliability (ICC = 0.92, Cronbach's α = 0.87) [31]. A total score of 76 and above is interpreted as fatigued for healthy adults [42].

*Visual Analog Scale:* The VAS has been shown to be a reliable outcome measure (ICC = 0.66, 95% CI = 0.39–0.83) for assessing the severity of fatigue [32]. In the present study, physical fatigue (VAS -PF) and mental fatigue (VAS-MF) were assessed using 100 mm-VAS, with a high value indicating a high level of fatigue. The scores for VAS-PF and VAS-MF were summed to obtain a total fatigue score (VAS) [43].

*Nottingham Health Profile:* The NHP measures the health-related quality of life and the impact of the individual's problems on the functions of her/his social roles [33]. The first part of the scale, consisting of 38 items, measures the individual's quality of life; the second part, consisting of 6 items, measures the impact of the individual's problems on her/his social roles. The first part which has six subscales including physical mobility, sleep, pain, energy level, emotional reactions, and social isolation was used in this study. The score for each subscale ranges from 0–100, and the total score is calculated by the sum of the scores of the six subscales. Higher scores indicate poorer quality of life [33]. The Turkish version of the scale is found to be reliable (r = 0.70–0.92, Cronbach's α = 0.56–0.83) and valid [33].

*Beck Depression Scale:* The 21-item scale assesses the severity of physical, emotional, motivational, and cognitive depressive symptoms experienced in the past week [34]. The total score ranges from 0 to 63, with a score of 0–3 for each item. High scores indicate increased severity of depressive symptoms [34]. Meites et al. reported that individuals with a score of 21 and above had severe depressive symptoms [44]. The Turkish version of the BDS has shown acceptable measurement properties (Cronbach's α = 0.80, r = 0.50) [34].

*Pittsburg Sleep Quality Index:* This 18-item scale assesses sleep quality in the past month. The total score range is 0 to 21 and the higher scores indicate poorer sleep quality [45]. The Turkish version of the scale has shown acceptable measurement properties [35].

### Statistical analysis

Statistical analysis was performed using MedCalc version 19.2.6 (MedCalc Software Ltd., Ostend, Belgium), SPSS version 26.0, and AMOS version 23.0. Participant characteristics are presented as means/standard deviations (SD) and medians/ 25%-75% for numerical data, and counts/percentages for categorical data. The normal distribution of continuous variables was tested using visual (histogram and probability plots) and analytic methods (Kolmogorov–Smirnov test). Nonparametric tests (Spearman correlation coefficient and Mann–Whitney's U test) were used because the variables were not distributed normally. The level of statistical significance for all inferential analyses was set at *p* < 0.05.

### Reliability

Test–retest reliability of the CFS and the subscales was examined using intraclass correlation coefficient (ICC) and 95% confidence interval based on a single measure and a 2-way mixed effects model with absolute agreement. The ICC value of < 0.50 indicates poor reliability, 0.50–0.75 indicates moderate reliability, 0.75–0.90 indicates good reliability, and > 0.90 indicates excellent reliability [28]. Test–retest reliability for the items of the CFS was examined using weighted kappa (κ) and 95% confidence interval. The weighted kappa value ≤ 0.20 indicates slight reliability, 0.21- 0.40 indicates fair reliability, 0.41–0.60 indicates substantial reliability, and 0.81–1.00 indicates almost perfect reliability [46]. Internal consistency was determined with Cronbach's α coefficient and item-total score correlations. The level of the correlation coefficient for the item-total correlation was interpreted as negligible (< 0.20), low (0.20–0.40), moderate (0.40–0.70), high (0.70–0.90), and very high (> 0.90) [47, 48]. The Cronbach's α value of ≥ 0.70 is considered acceptable, 0.80 good, and < 0.60 poor or unacceptable [49].

#### Measurement error

Measurement error was calculated by the smallest detectable change with 95% confidence (SDC_95_) based on the standard error of measurement (SEM) using the test–retest reliability statistics with the following formula [50]:

$$\begin{gathered} {\text{SEM}} = {\text{SD}} \times \sqrt {\left( {1 - ICC} \right)} \quad ({\text{SD:}}\,{\text{standard}}\,{\text{deviation}}\,{\text{of}}\,{\text{test}}\,{\text{and}}\,{\text{retest}}\,{\text{data}}) \hfill \\ {\text{SDC}}_{95} = {\text{SEM}} \times 1.96 \times \surd 2 \hfill \\ \end{gathered}$$

### Criterion validity

Predictive validity was measured using the receiver operating characteristic (ROC) curve and the area under the ROC curve (AUC). Participants were divided into two groups using the CIS cut-off score to determine whether the CFS discriminated between non-fatigued (group-non-fatigued) and fatigued (group-fatigued) participants [42]. An AUC value of 0.50 represents non-sensitivity, while a value of 1.00 represents perfect sensitivity and specificity [27]. The AUC value of at least 0.70 is recommended to show adequate validity of a scale [27]. The appropriate cut-off value of the CFS was determined through the Youden index (*J*) method (*J* = sensitivity + specificity − 1) [51].

### Construct validity

Factor analysis and hypothesis testing were used to assess construct validity.

*Factor Analysis:* The construct validity of the CFS was assessed with exploratory and confirmatory factor analysis. First, exploratory factor analysis was conducted because the CFS was translated into a new language and tested on a new population in which the factor structure of the scale had not yet been tested [52]. A confirmatory factor analysis was then conducted to verify the factor structure identified in the exploratory factor analysis [52].

The Kaiser–Meyer–Olkin measure of sampling adequacy (the value must be greater than 0.50) and Bartlett's test for sphericity (*p* value must be less than 0.05) were performed to determine whether the data and sample were suitable for exploratory factor analysis [53]. The analysis was performed using principal component analysis with varimax rotation [52] since varimax rotation was preferred over oblimin rotation in previous studies [11, 12, 39]. Factors were selected based on eigenvalues greater than one [54]. Factor loadings were considered meaningful if they were greater than 0.40, and it was accepted that factors should explain at least 50% of the total variance [26]. After the exploratory factor analysis, we hypothesized that the second-order 2-factor structure was appropriate for validating the dimensional structure of the scale. Then, the hypothesized factor structure was tested with the confirmatory factor analysis. The second-order 2-factor structure model was evaluated using the maximum likelihood method. Model fit was examined using the criteria of the following goodness-of-fit indices: (1) chi-square/degree of freedom ratio (CMIN/df) of ≤ 5.0, (2) significant chi-square result (*p* < 0.05), (3) comparative fit index (CFI) of ≥ 0.95, (4) standardized root mean square residual (SRMR) of ≤ 0.08, (5) non-normed fit index (NNFI) of ≥ 0.90, (6) goodness-of-fit index (GFI) of ≥ 0.90, and (7) root mean square error of approximation (RMSEA) of ≤ 0.08 [26, 55–57]. A model modification was performed based on the modification index with respect to the standardized expected parameter change [58].

*Hypothesis Testing:*

Hypothesis testing examined convergent and known-group validity by testing a total of 14 predefined hypotheses. The hypotheses were defined before data collection to prevent statistical bias in the evaluation of the results of the hypotheses. Rejection of less than 25% of hypotheses indicates good construct validity [27].

Convergent validity was assessed using 11 predefined hypotheses (Table 6) that examined the relationship between the CFS and other measurement instruments (CIS, VAS, NHP, BDS, PSQI) using Spearman correlation coefficient (r). The level of correlation coefficients was interpreted as negligible (< 0.20), low (0.20–0.40), moderate (0.40–0.70), high (0.70–0.90), and very high correlation (> 0.90) [47, 48]. The hypotheses were based on knowledge from research literature [13, 14, 38, 41, 43, 59]. These measures were chosen because the relationships between the CFS and the scales have been shown in the literature.

Known-group validity of the CFS was determined using 3 predefined hypotheses designed to investigate whether the scale could differentiate fatigue levels between male and female groups. Females are expected to have higher levels of fatigue based on research literature [15, 60, 61]. The known group validity was assessed using Mann–Whitney's U test. The predefined hypotheses were as follows:

- H1: The female group has significantly higher CFS scores than the male group.
- H2: The female group has significantly higher CFS-PF scores than the male group.
- H3: The female group has significantly higher CFS-MF scores than the male group.

## Results

### Cross-cultural validity

The researchers and translators did not encounter any problems during the translation process. The expert committee reached a consensus and confirmed the items as adequate in accordance with the following equivalences: semantic, idiomatic, experiential, and conceptual.

### Characteristics of the participants and normative data

Sociodemographic data and the results of outcome measures are shown in Table 1. The prevalence of fatigue was found 27.7% with a cut-off score of 4 points (bi-modal scoring) and 39.5% with a cut-off score of 12 points (Likert scoring). The mean (SD) CFS score was 12.2 (4.8) and 9.4 (4.6) for female and male participants, respectively. Female participants had higher scores than male participants obviously. There were no marked changes in values with increasing age. Normative data for the CFS and its subscales by sex and age are presented in Table 2.

**Table 1:** Sociodemographic and outcomes data of the participants

	Test group (n = 476)		Re-test group (n = 161)	
**Age** (year), mean (SD)	28.75 (5.8)		28.0 (5.4)	
**Gender,** n (%)				
Male	264 (55.5)		94 (58.4)	
Female	212 (44.5)		67 (41.6)	
**Body mass index** (kg/m^2^), mean (SD)	23.75 (3.2)		23.60 (3.3)	
**Marital status**, n (%)				
Married	173 (36.3)		56 (34.8)	
Single/divorced/widowed	303 (63.7)		105 (65.2)	
**Type of employment,** n (%)				
White-collar employment	186 (39.1)		56 (34.8)	
Blue-collar employment	204 (42.8)		76 (47.2)	
Unemployed/student	86 (18.1)		29 (18.0)	
**Hours of working in a week,** mean (SD)	39.39 (16.7)		36.39 (17.9)	
**Education,** n (%)				
Primary/secondary school	34 (7.1)		21 (13.1)	
High school	134 (28.2)		58 (36.0)	
Bachelor’s degree ≤	308 (64.7)		82 (50.9)	

Outcome measurements	Mean (SD)	Median (25%-75%)	Mean (SD)	Median (25%-75%)
**CFS** (0–33)	10.7 (4.9)	11 (7–14)	10.4 (4.9)	11 (6–14)
Physical fatigue (0–21)	7.3 (3.7)	7 (5–10)	7.2 (3.7)	7 (4–10)
Mental fatigue (0–12)	3.3 (1.8)	4 (2–4)	3.2 (1.7)	4 (2–4)
**CIS** (20–140)	71.5 (19.2)	74 (57–84.5)	73.7 (18.5)	79 (63–85)
Fatigue severity (8–56)	31.6 (10.0)	33 (24–39)	32.7 (9.6)	34 (27–39)
Concentration (5–35)	18.1 (5.8)	18.5 (14–22)	18.7 (5.7)	20 (14.5–23)
**VAS** (mm) (0–200)	82.15(43.7)	83 (45 -119)	77.8 (41.6)	71 (45–108)
Physical (mm) (0–100)	38.3 (22.7)	38.5 (20–56)	38.1 (21.0)	40 (20–55)
Mental (mm) (0–100)	43.8 (25.7)	46 (20–66)	39.7 (25.4)	36 (15–60)
**NHP** (0–100)	15.2 (14.3)	11.6 (2.1–23)	17.8 (15.2)	16.5 (4.4–26.9)
**BDS** (0–63)	6.8 (5.8)	6 (2–10)	6.8 (5.6)	6 (2–10)
**PSQI** (0–21)	4.9 (2.9)	5 (3–7)	4.7 (2.8)	4 (2–6)

SD: standard deviation, kg/m^2^: kilogram / square meter, CFS: Chalder Fatigue Scale, CIS: Checklist Individual Strength, VAS: Visual Analog Scale, mm: millimeter, NHP: Nottingham Health Profile, BDS: Beck Depression Scale, PSQI: Pittsburgh Sleep Quality Index

**Table 2:** Normative data of the CFS by age and gender

	X (SD)	M (25–75%)	X (SD)	M (25–75%)	X (SD)	M (25–75%)
**Female**	20–29 age (n = 126)		30–40 age (n = 86)		20–40 age (n = 212)	
CFS-PF	8.6 (3.9)	8 (6–12)	8.5 (3.6)	8 (6–11)	8.6 (3.8)	8 (6–11.5)
CFS-MF	3.8 (7.7)	4 (2–5)	3.6 (1.9)	4 (2–5)	3.7 (1.8)	4 (2–5)
CFS	12.3 (4.9)	12 (9–16)	12 (4.8)	12 (8–15)	12.2 (4.8)	12 (9–16)
**Male**	20–29 age (n = 160)		30–40 age (n = 104)		20–40 age (n = 264)	
CFS-PF	6.3 (3.4)	7 (4–8)	6.4 (3.3)	7 (4–8)	6.3 (3.4)	7 (4–8)
CFS-MF	3.2 (1.9)	3 (2–4)	2.9 (1.7)	3.5 (1–4)	3.1 (1.8)	3 (1–4)
CFS	9.5 (4.7)	10 (6–12)	9.3 (4.6)	11 (5–11.5)	9.4 (4.6)	10 (6–12)
**Overall**	20–29 age (n = 286)		30–40 age (n = 190)		20–40 age (n = 476)	
CFS-PF	7.3 (3.8)	7 (4–10)	7.4 (3.6)	7 (5–9)	7.3 (3.7)	7 (5–10)
CFS-MF	3.4 (1.8)	4 (2–4)	3.2 (1.9)	4 (1–4)	3.3 (1.8)	4 (2–4)
CFS	10.7 (5)	11 (7–14)	10.6 (4.8)	11 (7–14)	10.7 (4.9)	11 (7–14)

X: Mean, SD: standard deviation, M: Median, 25%-75%: percentiles, CFS-PF: Chalder Fatigue Scale-Physical Fatigue, CFS-MF: Chalder Fatigue Scale-Mental Fatigue, CFS: Chalder Fatigue Scale

### Reliability

Test–retest analysis was performed with 161 healthy young adults. Participants' demographic and socioeconomic characteristics were similar in the test and retest groups. The distribution of demographic and socioeconomic characteristics of participants is shown in Table 1. Test–retest reliability analysis revealed that the CFS-PF had good reliability, the CFS-MF had moderate reliability, and the CFS had good reliability. The ICC values with 95% confidence intervals were 0.76 (0.67–0.82), 0.67 (0.55–0.76), and 0.76 (0.68–0.83) for the CFS-PF, the CFS-MF, and the CFS, respectively. Item 2 and item 7 had the lowest weighted kappa value, while item 6 had the highest value. The results of the test–retest analysis are shown in Table 3.

**Table 3:** Test–retest reliability and internal consistency of the Chalder Fatigue Scale

	κ	ICC	95% CI		Cronbach’s α if item deleted	r
			Lower	Upper		
1. Do you have problems with tiredness?	0.33		0.20	0.44	0.848	0.60
2. Do you need to rest more?	0.21		0.10	0.32	0.856	0.48
3. Do you feel sleepy or drowsy?	0.32		0.20	0.45	0.846	0.63
4. Do you have problems starting things?	0.22		0.15	0.33	0.847	0.61
5. Do you lack energy?	0.40		0.29	0.51	0.840	0.70
6. Do you have less strength in your muscles?	0.45		0.33	0.56	0.847	0.61
7. Do you feel weak?	0.21		0.10	0.32	0.842	0.67
CFS-PF			0.67			
8. Do you have difficulty concentrating?	0.22		0.10	0.34	0.848	0.60
9. Do you make slips of the tongue when speaking?	0.42		0.29	0.55	0.863	0.39
10. Do you find it more difficult to find the correct word?	0.33		0.20	0.46	0.858	0.46
11. How is your memory?	0.34		0.20	0.49	0.863	0.37
CFS-MF		0.67	0.55	0.76		
CFS			0.68	0.83		

κ: Weighted kappa coefficient, ICC: intraclass correlation coefficient, CI: Confidence interval, r: Pearson correlation coefficient for item-total correlation, CFS-PF: Chalder Fatigue Scale-Physical Fatigue, CFS-MF: Chalder Fatigue Scale-Mental Fatigue, CFS: Chalder Fatigue Scale

The CFS-PF had good internal consistency, the CFS-MF had acceptable internal consistency, and the CFS had good internal consistency. The Cronbach's α-values were 0.862, 0.704, and 0.863 for the CFS-PF, the CFS-MF, and the CFS, respectively. Item-total correlations ranged from 0.37 to 0.70, and Cronbach's α values ranged from 0.840 to 0.863 when an item was deleted. Deletion of the items did not increase Cronbach's alpha of the scale; therefore, no item was omitted. The reliability analyses of the CFS are shown in Table 3.

#### Measurement error

The SEM and SDC_95_ values were 1.75 and 4.85 for the CFS-PF, 0.95 and 2.62 for the CFS-MF, and 2.30 and 6.38 for the CFS, respectively.

### Criterion validity

**Fig. 3:**
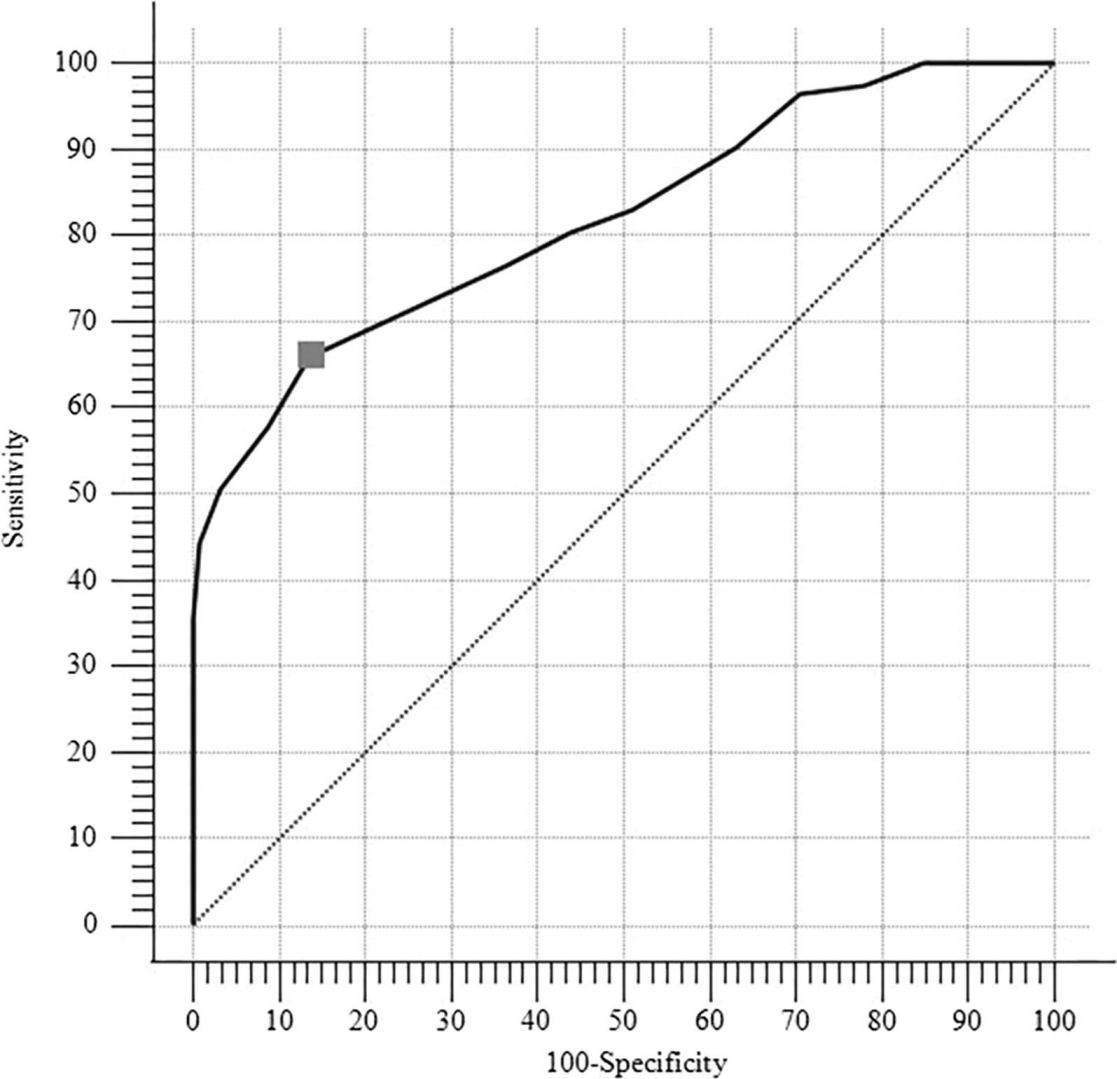
The receiver operating characteristic curve for the cut-off values of the Chalder Fatigue Scale (grey square: The optimal cut-off point was set at ≥ 12 with a sensitivity of 68.6% and a specificity of 82.5%)

*Predictive validity*: Group-non-fatigued (CIS < 76) included 242 participants and group-fatigued (CIS ≥ 76) included 234 participants. Group non-fatigued had a mean (SD) CFS score of 7.99 (3.62) and group-fatigued had a mean CFS score of 13.4 (4.52). A significant difference was found between group-non-fatigued and group-fatigued (*p* < 0.001). The AUC value was 0.817 (95% CI 0.779–0.851). The ROC curve is shown in Fig. 3. The optimal cut-off point for the CFS was set at ≥ 12 with a sensitivity of 65.8% (95% CI 59.3–71.9) and a specificity of 85.9% (95% CI 80.9–90.1). The criterion values of the ROC curve are shown in Table 4.

**Table 4:** Sensitivity and specificity values for different cut-off scores for the Chalder Fatigue Scale

Cut off score	Sensitivity	95% CI	Specificity	95% CI
≥ 10	80.34	74.7–85.2	55.79	49.3–62.1
≥ 11	76.50	70.5–81.8	63.22	56.8–69.3
**≥ 12**	**65.81**	**59.3**–**71.9**	**85.95**	**80.9**–**90.1**
≥ 13	57.69	51.1–64.1	90.91	86.6–94.2
≥ 14	50.43	43.8–57.0	96.28	93.1–98.3

CI: Confidence interval, bold indicates the optimal cut-off value

### Construct validity

*Factor analysis:* The results of the Kaiser–Meyer–Olkin and Bartlett's sphericity test indicated that the sample size was adequate (KMO = 0.838) and the items were appropriate (Bartlett's test of sphericity: χ^2^ = 2215.12, *p* < 0.001). Two factors were identified as a result of the analysis: Factor 1, physical fatigue (CFS-PF), and Factor 2, mental fatigue (CFS-MF) (Table 5). Seven items (items 1–7) were loaded onto Factor 1 (range 0.560–0.785), while four items (items 8–11) were loaded onto Factor 2 (range 0.430–0.867). Item 4 ("Do you have problems starting things?") and item 8 ("Do you have difficulty concentrating?") also loaded significantly on mental fatigue (0.432) and physical fatigue (0.477), respectively (Table 5). Using the same method, the unidimensionality of the subscales is shown in Table 5.

**Table 5:** Factor loadings for the items of the Chalder Fatigue Scale following principal component analysis with varimax rotation

The items of the CFS	Factor loadings (bifactor model of the CFS)		Factor loadings (unidimensional model of the subscales)	
	Factor 1 (physical fatigue)	Factor 2 (mental fatigue)	Factor 1^a^ (physical fatigue)	Factor 2^b^ (mental fatigue)
Item 1	**0.752**	0.086	**0.738**	
Item 2	**0.690**	0.011	**0.646**	
Item 3	**0.680**	0.268	**0.735**	
Item 4	**0.560**	*0.432*	**0.668**	
Item 5	**0.785**	0.216	**0.821**	
Item 6	**0.737**	0.159	**0.767**	
Item 7	**0.748**	0.237	**0.791**	
Item 8	*0.477*	**0.534**		**0.667**
Item 9	0.005	**0.863**		**0.819**
Item 10	0.091	**0.867**		**0.857**
Item 11	0.264	**0.430**		**0.544**
Eigenvalues	3.842	2.362	3.837	2.145
Explained variance	34.927	21.468		
Explained total variance	56.396		54.813	53.635

Bold indicates items loaded to factor 1 and factor 2, italic indicates items loaded on both factors

^a^Kaiser–Meyer–Olkin measure of sampling adequacy: 0.83, Bartlett’s test of sphericity: χ^2^ = 1507.28, *p* < 0.001

^b^Kaiser–Meyer–Olkin measure of sampling adequacy: 0.66, Bartlett’s test of sphericity: χ^2^ = 439.85, *p* < 0.001

**Fig. 4:**
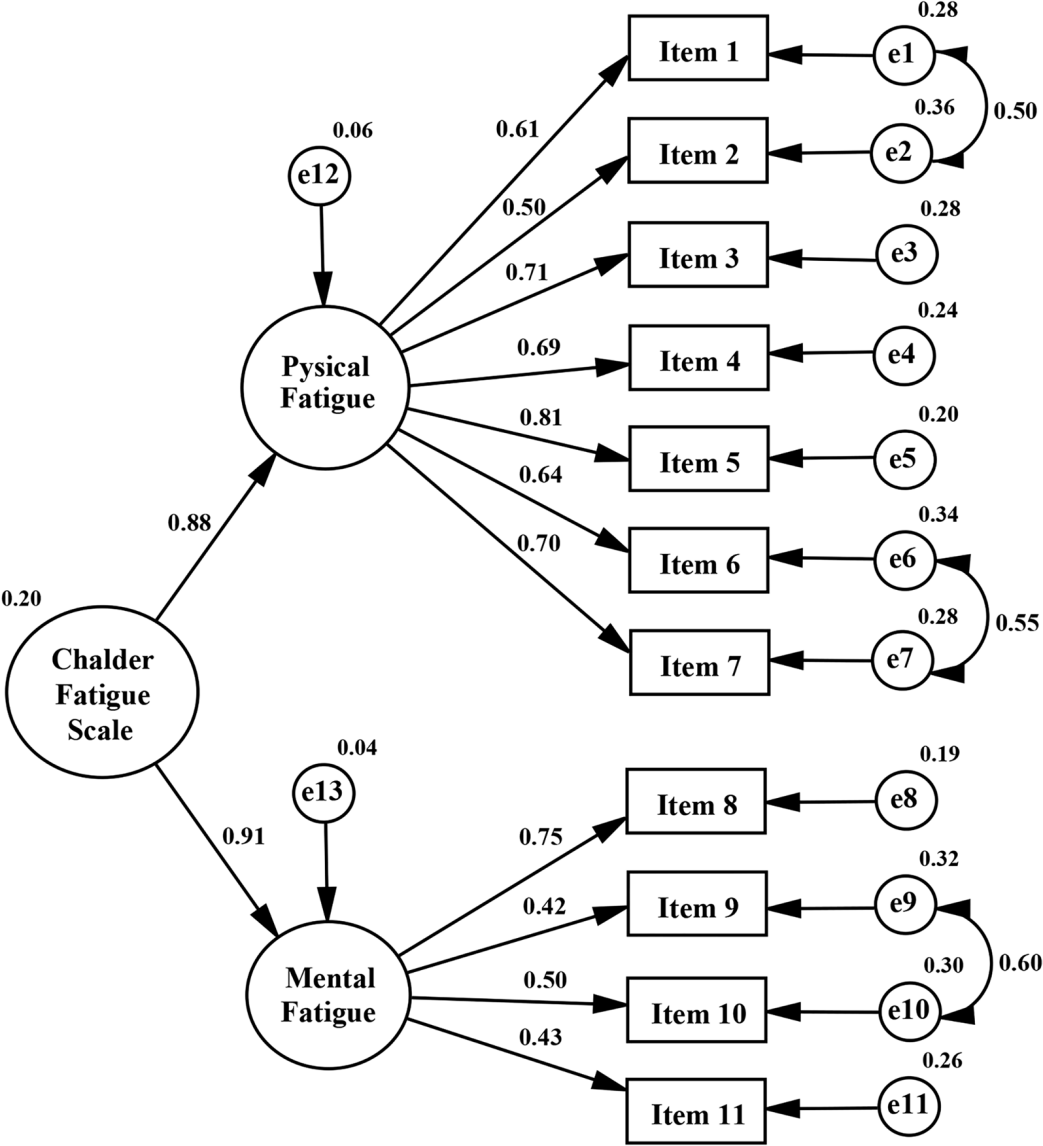
The second-order 2-factor model of the Chalder Fatigue Scale with standardized parameter estimates

After the two unidimensional factors were identified with exploratory factor analysis, the hypothesized dimensional structure of the scale (the second-order 2-factor) was validated by confirmatory factor analysis. Modifications were made to optimize the dimensional structure of the scale according to the modification indices, which suggested adding covariance between error items 1–2; 6–7; and 9–10. After the modifications, the second-order 2-factor model (Fig. 4) showed acceptable goodness-of-fit indices (CMIN/df: 3.03, *p* < 0.001, CFI:0.96, SRMR:0.02, NNFI: 0.95, GFI: 0.96, RMSEA: 0.06).

*Hypothesis testing*: Thirteen out of 14 predefined hypotheses (92.9%) were confirmed for the CFS and the subscales. Table 6 shows the results of the convergent validity analysis according to predefined hypotheses. The CFS had a moderate positive correlation with CIS and VAS, and a low positive correlation with BDS, NHP, and PSQI. The CFS-PF had a moderate positive correlation with CIS-FS and VAS-PF while a low positive correlation with BDS. The CFS-MF had a moderate positive correlation with CIS-C while a low positive correlation with VAS-MF and BDS. Overall female participants (20–40 age) had higher fatigue in the CFS-PF (*p* < 0.001), in the CFS-MF (*p* < 0.001), and in the CFS (*p* < 0.001) (Table 2).

**Table 6:** The results of the convergent validity analysis according to predefined hypotheses

	Hypothesized	Observed r	*P*-value	Interpretation	Confirmed/Refuted
CFS and CIS	Moderate positive (0.40–0.70)	+ 0.51	< 0.001	Good	Confirmed
CFS and VAS	Moderate positive (0.40–0.70)	+ 0.45	< 0.001	Good	Confirmed
CFS and NHP	Low positive (0.20–0.40)	+ 0.30	< 0.001	Good	Confirmed
CFS and BDS	Low positive (0.20–0.40)	+ 0.31	< 0.001	Good	Confirmed
CFS and PSQI	Low positive (0.20–0.40)	+ 0.31	< 0.001	Good	Confirmed
CFS-PF and CIS-FS	Moderate positive (0.40–0.70)	+ 0.55	< 0.001	Good	Confirmed
CFS-PF and VAS-PF	Moderate positive (0.40–0.70)	+ 0.46	< 0.001	Good	Confirmed
CFS-PF and BDS	Low positive (0.20–0.40)	+ 0.31	< 0.001	Good	Confirmed
CFS-MF and CIS-C	Moderate positive (0.40–0.70)	+ 0.41	< 0.001	Good	Confirmed
CFS-MF and VAS-MF	Moderate positive (0.40–0.70)	+ 0.21	< 0.001	Insufficient	Refuted
CFS-MF and BDS	Low positive (0.20–0.40)	+ 0.24	< 0.001	Good	Confirmed

r: Spearman correlation coefficient, CFS: Chalder Fatigue Scale, CIS: Checklist Individual Strength, VAS: Visual Analog Scale, NHP: Nottingham Health Profile, BDS: Beck Depression Scale, PSQI: Pittsburg Sleep Quality Index, CFS-PF: Chalder Fatigue Scale-Physical Fatigue, CIS-FS: Checklist Individual Strength- Fatigue severity, VAS-PF: Visual Analog Scale- Physical Fatigue, CFS-MF: Chalder Fatigue Scale-Mental Fatigue, CIS-C: Checklist Individual Strength-Concentration, VAS-MF: Visual Analog Scale- Mental Fatigue

### Power analysis

A post-hoc power analysis was conducted using R version 4.2.1 (packages ‘semPower’ and ‘ICC.sample.size’, R Core Team, 2022) to determine the exact power of the present study. The post-hoc power for the confirmatory factor analysis (alpha = 0.05, degrees of freedom: 40, n = 476, null hypothesized RMSEA value of 0.08, and alternative hypothesized RMSEA value of 0.06) and the ICC (obtained ICC = 0.67, null hypothesized ICC = 0.50, number of ratings:2, alpha = 0.05, two-tailed, n = 161) were 80.32% and 91.1%, respectively. Results show that the achieved power was sufficient to draw valid and reliable conclusions based on obtained data.

## Discussion

Fatigue is a worldwide problem that significantly affects an individual's physical, cognitive, emotional, or social abilities. In this study, the cross-cultural adaptation of the CFS into Turkish was conducted and its psychometric properties were investigated in healthy adults. The results of the study show that the Turkish CFS has a 2-factor structure and the scale and its subscales have strong measurement properties that make it a reliable and valid instrument for research and practice. The present study also established normative data of the CFS for healthy young adults by sex and age to determine the expected prevalence and comorbidity in a patient population.

Culture may have an impact on fatigue through differences in culture-specific lifestyle and norms of illness behavior [62, 63]. The prevalence of fatigue in Lausanne (Switzerland) middle-aged population (age range 45–86 years) was reported to be 22.1% as measured by the Fatigue Severity Scale at a score of ≥ 4 points [64], whereas Lerdal et al., reported 46.7% in the Norwegian population aged 19–82 years with the same outcome measure [65]. The increased prevalence was explained by the younger study population. In a study conducted among general practice registered individuals aged 18–45 years, a prevalence of fatigue of 38% was reported using the CFS [61]. In men, the mean fatigue score was 24.1 (95% CI 24–24.2) and in women, 25.2 (95% CI 25.1–25.3), increasing slightly with age [61]. However, Loge et al. reported a lower prevalence of 22% in the general population aged 19–80 years as measured by CFS (bi-modal scores of ≥ 4 points) with lower scores in both genders [15]. Female participants had the CFS scores of 12.3 and 12.5, and male participants had the scores of 11.1 and 11.5 at ages < 29 and 30–39, respectively. In the present study, the prevalence of fatigue was 27.7% at a cut-off score of 4 points (bi-modal scoring) and 39.5% at a cut-off score of 12 points (Likert scoring). The mean CFS scores for female participants were 12.3 and 12, and for male participants were 9.5 and 9.3 at similar age groups. Male participants had relatively lower CFS scores in Turkish adults compared with the Norwegian population [15]. Female participants had significantly higher scores than male participants in both studies. Loge et. al. reported positive correlations with age in both genders with the highest scores occurring in individuals aged 60 years or older [15]. In addition, recent studies have shown an increased prevalence of fatigue (46–52%) measured with CFS during the COVID-19 pandemic in different countries in population-based cohorts [7, 66]. The use of such instruments is necessary to compare with the norms of the general population to facilitate the interpretation of fatigue scores. Further studies are needed to establish a normative database for different age groups in the clinical and healthy Turkish population.

Test–retest reliability is a measure of reliability determined by administering the same test twice over a period of time. Test–retest reliability of the CFS was examined in the Japanese version, which was administered to 52 healthy children aged 11–13 years. The reliability of the scale was found to be moderate (ICC = 0.55) [16]. In the Turkish CFS, the ICC values with 95% confidence intervals were 0.76 (0.67–0.82), 0.67 (0.55–0.76), and 0.76 (0.68–0.83) for the CFS-PF, the CFS-MF, and the CFS, respectively. The weighted kappa values of the items ranged from 0.21 to 0.45, with the item 2 and the item 7 having the lowest weighted kappa value and the item 6 having the highest weighted kappa value. The overall scale and its subscales proved to be reliable in this study. Previous studies have not considered the test–retest reliability of the CFS in adults; hence the results of this study may add value to the research literature.

The internal consistency of a scale can be assessed by the analysis of the item-total score correlation and Cronbach's α. The item-total score correlation tests the homogeneity of a scale. The CFS is found to have low to moderate item-total correlations in the present study (0.36 < r < 0.71). However, compared to our study, the Norwegian version of the scale showed lower item-total correlations (0.11 < r < 0.66) [15]. Another indicator of the internal consistency of a scale is Cronbach's α coefficient. In the original study, the CFS-PF, the CFS-MF, and the CFS were found to have good internal consistency among individuals registered in general practice (Cronbach's α = 0.86, 0.82, and 0.89, respectively) [12]. The other versions of the scale also showed acceptable to good internal consistency (Cronbach's α = 0.70–0.89) [11–13, 36]. Consistent with previous studies, the Turkish CFS showed acceptable to good internal consistency in adults (Cronbach's α = 0.862, 0.704, and 0.863, respectively). Additionally, the measurement error of the scale was further analyzed. Minimal clinical significance for the overall CFS score was reported to be ≥ 9 points in patients with chronic fatigue syndrome [67]. In the present study, the measurement error was found to be 7 (6.38) points in healthy adults.

Criterion validity compares the responses of a new measurement with those of other, better-established instruments (concurrent validity) or a future standard (predictive validity). The predictive validity of the original CFS was examined using ROC analysis and showed good performance in distinguishing patients with chronic fatigue syndrome from the general population [12]. A score of 29 points and above was established as the cut-off value for chronic fatigue syndrome [12]. Similarly, in the present study, a ROC curve analysis was used to evaluate the ability of the scale to discriminate between non-fatigued and fatigued healthy individuals. The CFS cut-off value was found to be ≥ 12 points, which demonstrated a sensitivity of 65.8% and a specificity of 85.9%. The ROC curve analysis showed acceptable accuracy.

Exploratory factor analysis revealed that the scale consists of two factors including physical and mental fatigue in the general population [11, 12, 15], in primary care patients [36], and fatigued patients [37]. Similar to the aforementioned studies, in this study, two factors were extracted for healthy adults, including physical fatigue and mental fatigue. The scale was found to have clear item loading for both fatigue subscales. The first factor, which was labeled "physical fatigue," primarily included physical exhaustion related to feeling tired. The second factor, which was labeled "mental fatigue," included items primarily questioning the person's cognitive activity. Furthermore, consistent with the literature [11, 12, 36, 37], item 4 loaded slightly but significantly on the mental fatigue factor and item 8 loaded slightly but significantly on the physical fatigue factor. These results suggest that slight changes to these items may contribute to the factor structure of the scale. Therefore, we suggest revising item 4 as follows: "Do you have difficulty starting something physical?" to emphasize the "physical" aspect of fatigue and item 8 as follows: " Do you have difficulty focusing your attention?” to emphasize the "mental" aspect.

In this study, the 2-factor model for healthy young adults identified by exploratory factor analysis was also demonstrated by confirmatory factor analysis. In line with the present study, in the general Chinese population [13] and university students [38], confirmatory factor analysis of the CFS revealed a two-factor structure identified by exploratory factor analysis. However, in the study by Fong et al. three factors were extracted, including physical fatigue (items 1–3), low energy (items 4–7), and mental fatigue (items 8–11) in the Chinese general population, using exploratory structural equation modeling [59]. Similarly, a 3-factor structure was found in university students in the Korean version of the scale [14]. Different study populations and different analysis methods may explain the differences in the observed structure of CFS between studies.

Regarding convergent validity, the predefined hypotheses were sufficiently confirmed by the study. In the Chinese version, the CFS was found to be moderately correlated with anxiety, depression (r = 0.54–0.68), and quality of life (r = 0.37–0.40) [13]. A moderate correlation was also found with depression, sleep, and quality of life in the Korean (r = 0.52–0.58) and Polish (r = 0.48–0.55) versions, respectively [14, 38]. Fong et al., however, found a low correlation with sleep quality (r = 0.21–0.30) and quality of life (r = 0.21–0.42) and a moderate correlation with depression (r = 0.32–0.46) [59]. In the present study, the CFS had a low correlation with quality of life (r = 0.30), depression (r = 0.31), and sleep quality (r = 0.31). The CFS-PF had a moderate positive correlation with the CIS-FS (r = 0.55) and VAS-PF (r = 0.46), and the CFS-MF had a moderate positive correlation with the CIS-C (r = 0.41) and a low positive correlation with VAS-MF (r = 0.21). Similarly, in the study by Worm-Smeitin, a moderate correlation between CFS-PF and CIS-FS (r = 0.439) and between CFS-MF and CIS-C (r = 0.506) was demonstrated [41]. Consequently, the CFS and its subscales appear to have good convergent validity. In addition, the known group validity analysis showed that the scale could determine differences between two independent groups. The severity of fatigue was found to be greater in women than in men, which is consistent with previous studies [15, 60, 61].

The study has some limitations that can be considered as recommendations for future research. Our respondents were selected from the community using a suboptimal sampling method (snowball sampling), which may limit the generalizability of the present results. In addition, the psychometric properties of the scale should be examined in clinical groups. Because different clinical groups may answer the questions differently, measurement invariance could be investigated in future studies.

## Conclusion

Fatigue is an important indicator of overall health in a variety of populations. In healthy individuals, fatigue negatively affects quality of life, sleep quality, and emotional well-being. Measurement of fatigue should be complementary to clinical assessments in order to select appropriate treatment options, and population-based normative data are needed to evaluate the effectiveness of these strategies. The results showed that the scale has good psychometric properties. The CFS seems to be a promising instrument to be used in different study populations for the assessment and management of fatigue.

## Data Availability

All data and materials support our claims and comply with field standards.

## Abbreviations

CFS: Chalder Fatigue Scale
COSMIN: COnsensus-based Standards for the selection of health Measurement Instruments
CFS-T: Chalder Fatigue Scale-Turkish
pCFS-T: Prefinal Turkish version of the Chalder Fatigue Scale
BDS: Beck Depression Scale
NHP: Nottingham Health Profile
SPSS: Statistical Package for Social Sciences
CIS: Checklist Individual Strength
VAS: Visual Analogue Scale
PSQI: Pittsburg Sleep Quality Index
ROC: Receiver operating characteristic
AUC: Area under the ROC curve
ICC: Intraclass correlation coefficient
SDC_95_: Smallest detectable change with 95% confidence
SEM: Standard error of measurement
95% CI: 95% Confidence interval
